# Dysregulation of Epigenetic Control Contributes to Schizophrenia-Like Behavior in Ebp1^+/−^ Mice

**DOI:** 10.3390/ijms21072609

**Published:** 2020-04-09

**Authors:** Inwoo Hwang, Jee-Yin Ahn

**Affiliations:** 1 Department of Molecular Cell Biology, Sungkyunkwan University School of Medicine, Suwon 16419, Korea; inwhwang@skku.edu; 2 Single Cell Network Research Center, Sungkyunkwan University School of Medicine, Suwon 16419, Korea; 3 Samsung Biomedical Research Institute, Samsung Medical Center, Seoul 06351, Korea

**Keywords:** erbB3 binding protein (EBP1), schizophrenia, hippocampus

## Abstract

Dysregulation of epigenetic machinery can cause a variety of neurological disorders associated with cognitive abnormalities. In the hippocampus of postmortem Schizophrenia (SZ) patients, the most notable finding is the deregulation of GAD67 along with differential regulation of epigenetic factors associated with glutamate decarboxylase 67 (GAD67) expression. As we previously reported, ErbB3-binding protein 1 (EBP1) is a potent epigenetic regulator. EBP1 can induce repression of Dnmt1, a well-studied transcriptional repressor of GAD67. In this study, we investigated whether EBP1 contributes to the regulation of GAD67 expression in the hippocampus, controlling epigenetic machinery. In accordance with SZ-like behaviors in *Ebp1*^(+/−)^ mice, heterozygous deletion of EBP1 led to a dramatic reduction of GAD67 expression, reflecting an abnormally high level of Dnmt1. Moreover, we found that EBP1 binds to the promoter region of HDAC1, which leads to histone deacetylation of GAD67, and suppresses histone deacetylase 1 (HDAC1) expression, inversely mirroring an unusually high level of HDAC1 in *Ebp1*^(+/−)^ mice. However, EBP1 mutant (p.Glu 183 Ter) found in SZ patients did not elevate the expression of GAD67, failing to suppress Dnmt1 and/or HDAC1 expression. Therefore, this data supports the hypothesis that a reduced amount of EBP1 may contribute to an etiology of SZ due to a loss of transcriptional inhibition of epigenetic repressors, leading to a decreased expression of GAD67.

## 1. Introduction

The ErbB3-binding protein 1 (EBP1)-related gene *Ebp1* (*Pa2g4*) (chromosome 12q13.2 in humans; chromosome 10 D3 in mouse) is comprised of 10 exons and encodes two alternatively spliced EBP1 isoforms, p48 and p42 [1]. The primary structure of two isoproteins, p48 (1-394 amino acids) and p42(55-394 amino acids), is different. Isoprotein p42 is missing 54 amino acids at its N-terminus. EBP1 isoforms have distinct functions and participate in various cellular processes. For instance, p48 EBP1 suppresses apoptosis and promotes cell proliferation, signaling as an oncoprotein through Akt activation and p53 degradation [2,3,4]. Whereas p42 EBP1, known as a binding partner of ERBB3 [5], can inhibit PI3K activity via p85-subunit degradation and recruitment of the E3 ligase and HSP70/CHIP complexes [6,7]. Despite distinctive cellular functions of two EBP1 isoforms during early embryonic development, only p48 EBP1 is detectable at both RNA and protein levels throughout the entire organism including brain tissues. EBP1 p48 isoform was found to be predominantly localized in neurons, rather than astrocyte, of the embryonic brain. EBP1 p42 expression was observed in mice after birth [8]. In the injured hippocampus, introduction of p48EBP1 by AAV2 delivery system resulted in notable regeneration of axon growth [8], implying that p48EBP1 may play an important role in processes of neural development and recovery. However, the physiological function of p48 EBP1 were not defined in developing brain tissues in vivo, because mammalian models of EBP1 deletion have not been developed. 

Recently, we abrogated *Ebp1* gene expression in mice using replacing of exons 6–10 coupled with the insertion of IRES-eGFP cassette downstream of the *Ebp1* STOP codon and *NeoR* gene. The procedure resulted in the complete deletion of both EBP1 isoforms [9]. Unfortunately, genetic ablation of *Ebp1* causes embryonic lethality with massive cell death. *Ebp1*^(−/−)^ mice demonstrated upregulation of Suv39H1-dependent H3K9 trimethylation and subsequent activation of DNMT1 associated with markedly increased global DNA methylation. EBP1 binds with the promoter region of *Dnmt1*, represses its expression, and inhibits promoter methylation of the DNMT1 targeted genes such as, *Survivin*. Reintroduction of AAV2-EBP1 significantly reduced neuron death and relived gene repression in the embryonic brain slices. This data implies that EBP1 is associated with regulation of epigenetic repressors during brain development. However, the physiological role of EBP1 has never been addressed in neurodevelopmental disorders. 

Schizophrenia (SZ) is a neurodevelopmental disorder the clinical features of which include impairments of perception, cognition, and motivation. Reduced hippocampal volume is one of the most robust brain abnormalities detected in SZ patients [10,11]. In postmortem brains of SZ patients, selective increases in DNMT1 mRNA expression were detected in γ-aminobutyric acid (GABA)-ergic cortical interneurons [12] and in hippocampus [13]. GABA neuron dysfunction was also observed during abnormal l regulation of HDAC1 and death-associated protein 6 (DAXX). GABA neurons located in the cortex and hippocampus of SZ patients indicated pathophysiological changes including a decrease in glutamate decarboxylase (GAD) 67 mRNA (also referred as GAD1) [14,15]. Associated with adverse synaptic function, dendritic morphological alterations were also identified in multiple brain regions including hippocampus of SZ patients [16,17]. 

Emerging evidences implicated dysfunction of GABAergic signaling in SZ and indicated that epigenetic activation of DNMT1 is associated with SZ dysfunctions [18]. Recently, SZ exome meta-analysis consortium (SCHEMA) (October 2019) have reported a loss of function and missense mutation of *Ebp1* gene. Accordingly, we found that the loss of EBP1 expression disrupted the normal regulation of epigenetic factors including DNMT1, resulting in aberrant brain organogenesis. Furthermore, mRNA level of GAD67 was greatly decreased in the absence of EBP1. Therefore, we aimed to investigate whether EBP1 is associated with molecular pathology of SZ. In this study, to avoid early lethality caused by the homozygous loss of *Ebp1*, we have used *Ebp1*^(+/−)^ mice and investigated SZ-related behavior in those animals. Our data indicates that SZ-like behavioral features of *Ebp1*^(+/−)^ mice could be attributed to impaired expression of GAD67 due to dysregulation of epigenetic repressors, Dnmt1 and HDAC1.

## 2. Results

### 2.1. Ebp1^(+/−^) Mice Demonstrate Deficits in the Hippocampus Development

**Figure 1:**
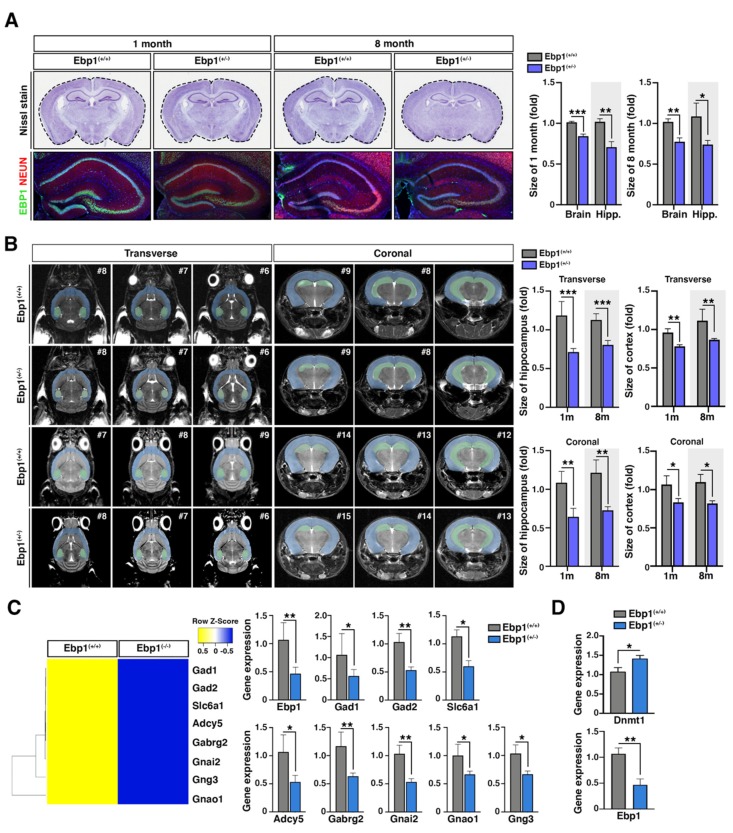
Deficits in hippocampus development in *Ebp1*^(+/−)^ mice. (**A**) *Ebp1*^(+/+)^ and *Ebp1*^(+/−)^ 1 month and 8 month old mouse brains were isolated and subjected to Nissl staining (to compare the brain sizes (upper)) or was stained with anti- ErbB3-binding protein 1 (EBP1) (red, marker for EBP1) and anti-NEUN (green, marker for neuronal cell) antibodies (to compare sizes of hippocampus (bottom)). The whole brain size of *Ebp1*^(+/−)^ mouse was smaller than that of *Ebp1*^(+/+)^ for about 15%. Especially, the hippocampus size was much smaller than that of Ebp1^(+/+)^ for about 30% 8 months after birth. Bar graphs display quantified data (right). * *p* < 0.05, ** *p* < 0.01, *** *p* < 0.001. scale bar: 500 μm. (**B**) Representative images of the whole brain structure of *Ebp1*^(+/+)^ and *Ebp1*^(+/-)^ mouse are shown; coronal (right) and transverse (left) T2-weighted images; MRI images of brain from 1 month (upper) or 8 month mice (bottom). Representative galleries of T2-weighted MRI images are shown. MRI was used to determine the size of hippocampus. The hippocampus (light green) and cortex (light blue) are highlighted. Bar graphs display quantified data (right). * *p* < 0.05, ** *p* < 0.01, *** *p* < 0.001. (**C**) The hierarchical clustering of differentially expressed RNA of GABAergic neuron gene set was derived using microarray of E12.5 Ebp1 deficient mouse brain samples. In the cluster heat map, red indicates high relative gene expression and green indicates low relative gene expression. To confirm down regulation of GABAergic neuron gene set, we performed qRT-PCR on hippocampus samples from 8-month-old Ebp1 WT and heterozygous mice. * *p* < 0.05, **p < 0.01. (**D**) RNA expression level of Dnmt1 (upper) and Ebp1 (bottom) were analyzed using qRT-PCR of hippocampus samples from 8-month-old *Ebp1*^(+/+)^ and *Ebp1*^(+/−)^ mice. The relative fold changes were quantified and shown in the bar graphs. * *p* < 0.05, ** *p* < 0.01. The mRNA levels were normalized to the levels of Gapdh. All data are shown as the mean ± s.e.m.

Homozygous *Ebp1*^(−/−)^ mice died on embryonic day (E) 13.5–15.5, displaying severe hemorrhages, deficient brain organogenesis, and reduced brain volume. Whereas heterozygous *Ebp1*^(+/−)^ mice were viable, although appeared smaller (approximately 28%, 1 week) and indicated changes in brain development. Heterozygous *Ebp1*^(+/−)^ mice retain half of the EBP1 mRNA and protein expression levels compared with wild type (WT) *Ebp1*^(+/+)^ mice. *Ebp1*^(+/−)^ mice were able to gain body mass comparable to that of WT mice at postnatal (P) day 60 [9]. The observed developmental brain defects were not recovered 8 months later, demonstrating smaller brain volume (18% and 22.5% at 1 month and 8 months respectively) and hippocampus (30% and 26% at 1 month and 8 months respectively) (Figure 1A). Consistently, the whole brain structure of *Ebp1*^(+/−)^ mice (P30) was investigated using magnetic resonance imaging (MRI). MRI analysis demonstrated an abnormality of hippocampal architecture and smaller hippocampus volume (35%) as well as a global reduction of cerebral volume (22%) compared to those of the WT mice. The reduced whole brain volume and the detected defects were not restored 8 months later (Figure 1B). 

A series of GABAergic genes including GAD67 were greatly decreased in microarray analysis data. The analysis was conducted using E13.5 brain lysates from homozygous *Ebp1*^(−/−)^ embryos that were compared with WT embryos. The qRT-PCR analysis indicated that GAD67 level was also significantly down regulated in heterozygous *Ebp1*^(+/−)^ mice at 8 months of age after birth, suggesting a dose-dependent regulation of these genes by EBP1 (Figure 1C). Primers for all target genes are listed in Supplementary Table S1. However, Dnmt1 mRNA level was still upregulated in heterozygous *Ebp1*^(+/−)^ mice (8 months after birth) (Figure 1D), confirming that the loss of EBP1 causes dysregulation of epigenetic control and unusually high level of Dnmt1 expression [9].

This data indicated that developmental defects caused by EBP1 deletion cannot be restored. Therefore, heterozygous *Ebp1*^(+/−)^ mice represent a useful model to study EBP1 postnatal function and reflect a dose-dependent relationship between EBP1 expression and epigenetic regulation in brain cells.

### 2.2. Impaired Neural Development of Stratum Oriens (SO) of CA3/2 Region in Hippocampus of Ebp1^(+/−)^ Mice

**Figure 2:**
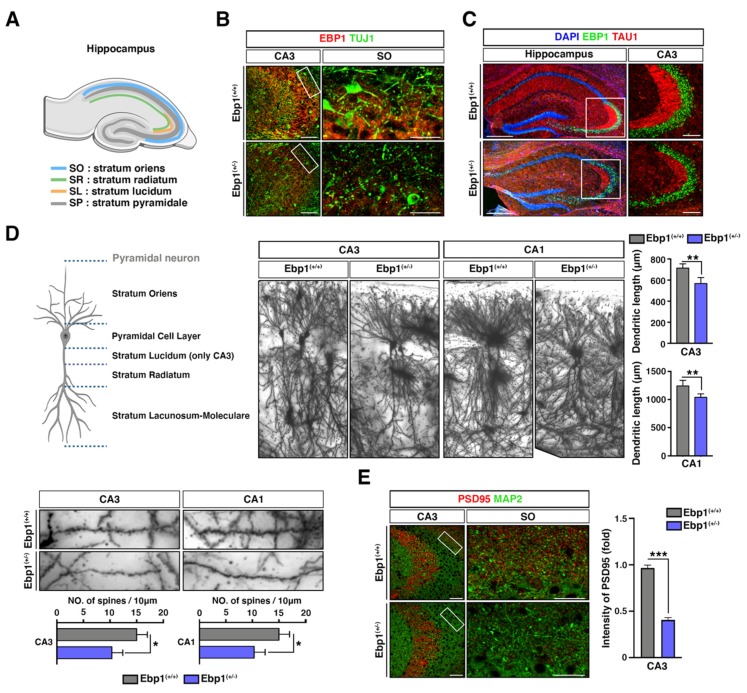
*Ebp1*^(+/−)^ mice displayed impaired neural development in stratum oriens (SO) of CA3/2 region in hippocampus. (**A**) representative hippocampus structure is displayed. Created with BioRender. (**B**) WT and *Ebp1* heterozygous mouse brain were isolated and subjected to IHC staining with anti-EBP1 and TUJ1 antibodies, early neuronal markers. CA3 region and cropped specific region as SO are shown. Scale bar: 200 μm. (**C**) Brain tissues were isolated from *Ebp1* WT and heterozygous mice and subjected to IHC analysis. The hippocampus was stained with anti-EBP1 (green) and TAU1 (red, axon specific marker) antibodies. The right-hand panel shows higher magnification of the CA3 region of the hippocampus; corresponding SO region is indicated by a white box. Intensity of Tau1 positive signals were quantified and displayed as bar graphs (right). Scale bar: 200 μm. (**D**) Fresh mouse brain tissues were stained with Golgi-Cox stain. Golgi-stained CA1 and CA3 hippocampal neurons (upper right) and their dendritic processes (bottom left) are shown, respectively. Total dendritic length was measured using image J. Dendrite numbers in the SO region of pyramidal neurons were counted within every 10 μm. * *p* < 0.05, ** *p* < 0.01. Created with BioRender. (**E**) Brain tissues, isolated from *Ebp1*^(+/+)^ and *Ebp1*^(+/−)^ mice, were stained with anti-PSD95 (red, marker for post-synapse) and MAP2 (green, marker for dendrites) antibodies. High power images show PSD95 and MAP2 double-labeling in the CA3 and corresponding SO regions (left). The intensity of PSD95 was quantified and shown as a bar graphs (right). *** *p* < 0.001. Scale bar: 200 μm. All data are shown as the mean ± s.e.m.

Stained with anti-Tuj1 antibodies, neuronal cells in hippocampus of Ebp1^(+/−)^ mice were found largely impaired in SO of CA3/2 region where dysregulation of GABAergic signaling was previously observed in postmortem samples from SZ patients [19] (Figure 2A,B). Moreover, stained with anti-Tau1 (an axon specific marker), neuronal cells showed decreased axon density in SO of CA3/2 region in hippocampus of Ebp1^(+/−)^ mice compared with that of WT mice (Figure 2C). This data indicates that loss of EBP1 could be involved in the regulation of GABAergic signaling linked to SZ. 

Considering that dendrites number undergoes substantial changes during development, abnormalities in these processes are likely to contribute the pathology of various brain disorders including SZ. Therefore, we questioned whether the loss of EBP1 is involved in the alteration of dendritic development in the hippocampus. Using Golgi impregnation method, we observed the greatly reduced number of dendrites (30% decrease) and reduced dendritic length (21% decrease) at both CA1 and CA3 regions in *Ebp1*^(+/−)^ mice compared with those of WT mice (Figure 2D). 

The reduction coincided with decreases in synaptic connections that are visualized using PSD95 staining intensity (Supplementary Figure S1), in SO of CA3/2 (Figure 2E). Consequently, our data implies that EBP1 is required for the dendritic development in hippocampal pyramidal neurons and, probably, contributes toward formation of the synaptic connections in this area.

### 2.3. Ebp1^(+/−)^ Mice Exhibit Schizophrenia-Like Behaviors

**Figure 3:**
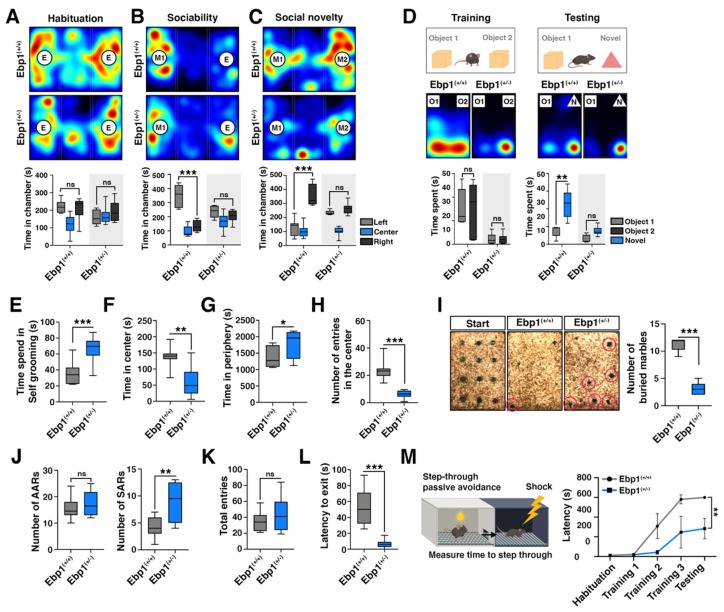
*Ebp1*^(+/−)^ mice display SZ-like behaviors. (**A**–**C**) Representative heat maps illustrating the time spent in different locations of the three chambers from the social preference test. (upper). (**A**) In the first 10 min, the test mouse was placed in the center and allowed to explore each chamber as habituation liberally. * *p* < 0.05. (**B**) In the second 10 min, an age- and gender-matched WT B6 mouse (mouse 1) that had never been exposed to the test mouse, was placed in the wired cage of left chamber. The other side remained empty. The test mouse was placed in the center of the cage and allowed to explore the chamber for 10 min liberally. * *p* < 0.05. (**C**) In the last experiment, a new stranger mouse (mouse 2) was introduced in the wired cage of right chamber. The test mouse was allowed to explore the chamber for 10 min liberally. * *p* < 0.05. E, empty; M1, mouse 1; M2, mouse 2. Ebp1^(+/+)^, *n* = 12; Ebp1^(+/+)^, *n* = 12. (**D**) Representative scheme and heat maps illustrating the time spent in different locations of novel object recognition test (upper). The test mouse was placed in the middle of objects and allowed to explore the cage and measured explore time with two objects for 10 min (bottom, left). Next day, one of the objects was replaced with a novel object and measured explore time with object1 and novel object for 10 min. ** *p* < 0.01. O1, object 1; O2, object 2; N, novel object. Ebp1^(+/+)^, *n* = 12; Ebp1^(+/+)^, *n* = 12. (**E**) Self-grooming assessed as repetitive grooming was scored during 10 min manually. * *p* < 0.05. Ebp1^(+/+)^, *n* = 12; Ebp1^(+/+)^, *n* = 12. (**F**,**G**) Open field test; the area was separated into two zones and included the center (28.5 × 28.5 cm) and the periphery (around of center zone) areas. * *p* < 0.05. (**H**) The number of entries in the center zone was counted. Open field test was performed for 20 min. Ebp1^(+/+)^, *n* = 12; Ebp1^(+/+)^, *n* = 12. (**I**) Marble-burying test; WT (*n* = 12) and mutant mice (*n* = 12) were placed in the cage for 30 min and allowed to roam liberally. The over 2/3 buried marbles were counted. * *p* < 0.05. (**J**–**L**) Y-maze experimental data; (**J**) Visiting of arms in the order 1–2-3 is an example of an alternation (left, AAR), whereas the order of 1–2-1 was considered as the non-alternation (right, SAR). (**K**) Total entry number and (L) latency to exit were counted. AAR, alternation arm returns; SAR, same arm returns. Ebp1^(+/+)^, *n* = 12; Ebp1^(+/+)^, *n* = 12. (**M**) Passive avoidance test; WT (*n* = 12) and *Ebp1* mutant mice (*n* = 12) were tested and the duration of the freezing time was checked (maximum trial duration = 600 s). Light was used as the conditioned stimulus. ** *p* < 0.01. All data are shown as the mean ± s.e.m.

To investigate whether EBP1 heterozygous mice can display abnormal behaviors comparable to SZ [20], we evaluated the level of social behavioral deficits including social withdrawal and isolation using three chambered assay of social approach and preference for social novelty [21,22]. Both *Ebp1*^(+/−)^ and WT mice demonstrated no preferences for two empty cages located in left and right chambers during the habituation period (Figure 3A). However, when a stranger (new) mouse was introduced to the cage in one of the chambers, WT mice spent more time in the mouse-containing chamber than in the empty cage-containing chamber and interacted more extensively with the new mouse and peers. In contrast, *Ebp1*^(+/−)^ mice showed no preferences and interests to interact with the new (freshly introduced) mouse and interacted less frequently with peer mice (Figure 3B). 

When a second stranger mouse was placed in the unoccupied side chamber to assess the discrimination between a new and a familiar mouse, WT mice spent significantly more time in the chamber and in close interactions with the second mouse than with the familiar mouse (introduced first). Alternatively, *Ebp1*^(+/−)^ mice displayed no preferences for the social novelty (Figure 3C).

To test main function of hippocampus for short-term memory, we performed novel object recognition test. *Ebp1*^(+/+)^ and *Ebp1*^(+/−)^ mice were replaced with two objects and spent time for 10 min. We found *Ebp1*^(+/−)^ mice spent less time in the objects (Figure 3D, left). Next day, those mice were replaced with one old object and one novel object, which is different color and shape. We found that short-term memory in the novel object recognition task was significantly impaired in the *Ebp1*^(+/-)^ group compared with the *Ebp1*^(+/+)^ control group (Figure 3D, right). We also found significantly increased levels of self-grooming rates in *Ebp1*^(+/−)^ mice who were engaged in the repetitive grooming task (Figure 3E). Collectively, these results suggest that *Ebp1*^(+/−)^ mice exhibit social behavioral deficits. 

In the open field test, *Ebp1*^(+/−)^ mice spent much less time in the center of the cage (Figure 3F) and stayed longer in the periphery (Figure 3G) compared with WT mice. Additionally, *Ebp1*^(+/−)^ mice dared to enter the cage center significantly less frequently compared to WT mice (Figure 3H), revealing anxiety-like behaviors and exploratory deficits. However, *Ebp1*^(+/−)^ mice did not show signs of hyperactivity and walked for slightly shorter travel distances (20 min observation frame) compared with WT mice (Supplementary Figure S2). This data suggests that hyperactivity might be affected by the anxiety level only. Furthermore, in the marble-burying test, *Ebp1*^(+/−)^ mice buried fewer marbles compared with WT mice, implying abnormal emotional behaviors of the mice (Figure 3I). 

Cognitive dysfunctions including working memory and learning deficits are well known characteristics of SZ [23,24]. To evaluate cognitive behavioral change in *Ebp1*^(+/−)^ mice, we used Y maze assessment. The test is frequently employed to estimate working memories [25,26] and passive avoidance learning, which depend on cortical neurons and hippocampus activities in rodents [27]. In the Y-maze test, *Ebp1*^(+/−)^ mice exhibited a reduction in the spontaneous alteration rate, although total entries were higher compared to WT mice (Figure 3J,K). Additionally, we observed that *Ebp1*^(+/−)^ mice were faster to exit the maze (Figure 3L), indicating that the working memory may be impaired in *Ebp1*^(+/−)^ mice. In the passive avoidance test, *Ebp1*^(+/−)^ mice showed the notably shorter latency response immediately after 2nd and 3rd foot shocks compared to WT mice (Figure 3M), indicating the reduced memory strength. On the next day, while WT mice showed a latency time of 600 s, *Ebp1*^(+/−)^ mice showed only 200 s latency time, reflecting that *Ebp1*^(+/−)^ mice failed to retain the memory and prolonged learning (Figure 3M). Conclusively, *Ebp1*^(+/−)^ mice exhibited SZ-like behaviors including the deficit in social interaction, the anxiety-like and abnormal emotional behaviors, and impaired learning and memory.

### 2.4. Increased DNMT1 Levels Alter GAD67 Promoter Methylation in Ebp1^(+/−)^ Mice

**Figure 4:**
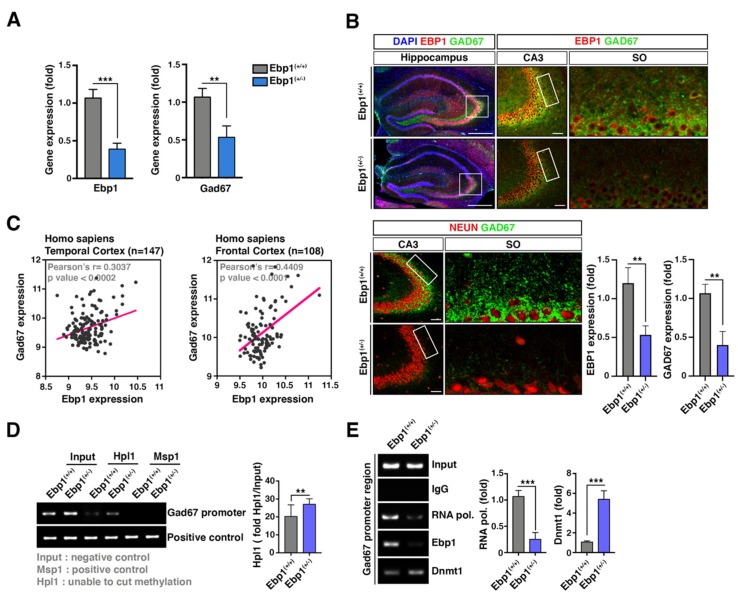
Increased DNMT1 levels in *Ebp1^(+/−)^* mice alters GAD67 promoter methylation. (**A**) Quantitative qRT-PCR analysis of gene expressions (Ebp1 and Gad67) was conducted in the samples from hippocampus of *Ebp1* WT and heterozygous mice. The relative fold changes were quantified and shown in the bar graphs. The expression levels were normalized using Gapdh. ** *p* < 0.01, *** *p* < 0.001. (**B**) Endogenous protein expression levels of GAD67 in hippocampus were measured using IHC with anti-EBP1 (red) and GAD67 (green) antibodies or anti-NEUN (red, neuronal cell marker) and GAD67 (green) antibodies EBP1 and GAD67 intensities were quantified and shown as bar graphs (right). Scale bar: 500 μm. (**C**) Scatterplots indicates the correlation analysis between Ebp1 and Hdac1 expression levels from temporal cortex (left) and frontal cortex (right). ** *p* < 0.01. (**D**) Genomic DNA (gDNA) was extracted from brain hippocampus of *Ebp1*^(+/+)^ and *Ebp1*^(+/−)^ using gDNA extraction kit (Sigma-Aldrich, Saint Louis, MO, USA). The gDNA was subjected to CG island-cut using enzymes Hpl1 (cut only non-methylation CG island) or Msp1 (cut CG island as positive control). The gDNA was assessed for methylation levels using the methylation-specific PCR with GAD67 promoter primer sets. ** *p* < 0.01. (**E**) MEF cells were subjected to ChIP assay with anti-mouse IgG as negative control, RNA polymerase II as positive control, and with EBP1 or DNMT1 antibodies. The ChIP assay was performed suing ChIP assay kit (cat. 17-259, Millipore, Temecula, CA, USA). The two purified DNAs were observed using qRT-PCR with specific primer set to amplify Gad67 promoter region. *** *p* < 0.001. All data are shown as the mean ± s.e.m.

One of the most highly replicated findings is the decreased GAD67 expression in a subset of GABAergic neurons investigated in postmortem brain studies in SZ patients. We found that *Ebp1*^(+/−)^ mice showed SZ-like behavioral deficits and impaired expression of a series of GABAergic genes including GAD67. Consequently, we aimed to test whether EBP1 loss is responsible for a decreased expression of GAD 67 in the hippocampus of *Ebp1*^(+/−)^ mice. Indeed, not only Gad67 mRNA levels, but also GAD67 protein expression were greatly decreased in the hippocampus of *Ebp1*^(+/−)^ mice, especially, in the SO region of CA3, compared to that of WT mice (Figure 4A,B). We also noticed this correlation in the brain cortex of SZ patients (Figure 4C). 

Recent evidence suggests that a reduced expression of GAD67 levels in postmortem SZ temporal cortex is associated with changes in the epigenetic regulation including hypermethylation [28]. For instance, significantly higher level of Dnmt1 was observed in the SZ samples. Escaping from EBP1-dependent transcriptional repression, Dnmt1 expression was unusually high in *Ebp1*^(+/−)^ mice [9]. Therefore, we questioned whether Dnmt1 mediates aberrant methylation of GAD67 promoter in the hippocampal neurons which may lead to upregulation of GAD67. We tested whether a decreased expression of GAD67 in *Ebp1*^(+/−)^ mice is caused by its promoter methylation associated with heterozygous deletion of Ebp1. Genomic DNA was extracted from the hippocampus, treated with a methylation-sensitive restriction enzymes, and followed by PCR. Consequently, the CpG methylation was evidently observed in the GAD67 promoter in *Ebp1*^(+/−)^ mice compared to WT mice (Figure 4D). 

The loss of Ebp1 caused upregulation of Dnmt1 and could lead to the GAD67 methylation. To confirm this suggestion, we performed a ChIP assay using anti-EBP1 antibodies in the hippocampus of *Ebp1*^(+/−)^ and WT mice. In the presence of half-reduced level of EBP1, Dnmt1 was found associated with GAD67 promoter. RNA polymerase II was weakly associated with GAD67 promoter compared to the WT (Fig 4E), indicating a reduced level of GAD67 expression observed in the hippocampus of *Ebp1*^(+/−)^ mice which could be caused by Dnmt1-mediated promoter methylation.

### 2.5. EBP1 Expresses HDAC1 Transcription and Enhances GAD67 Expression

**Figure 5:**
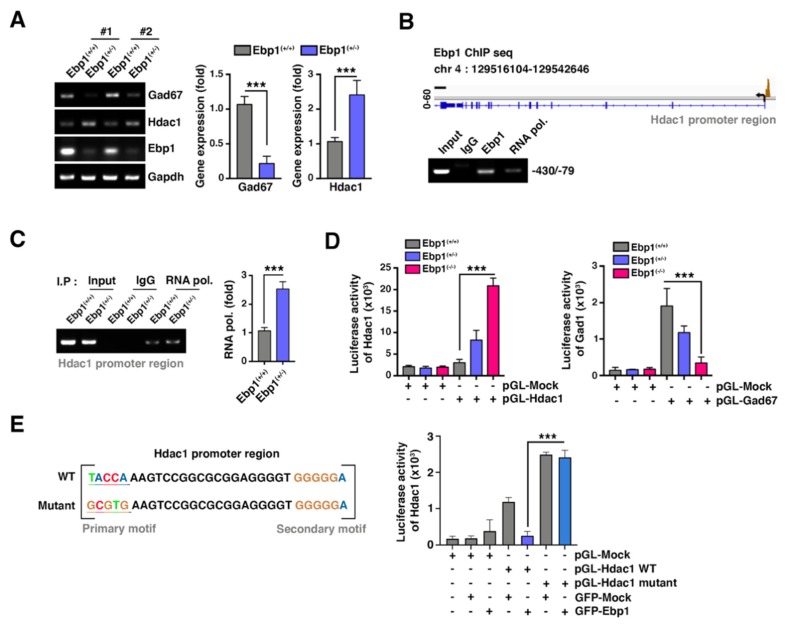
EBP1 repress histone deacetylase 1 (HDAC1) transcription and enhances Gad67 expression. (**A**) RNA expression levels of GAD67 and HDAC1 were measured using RT-PCR with samples of mouse hippocampus from *Ebp1*^(+/+)^ or *Ebp1*^(+/−)^ mice (left); RT-PCR quantification data is shown as bar graphs (right). *** *p* < 0.0001. (**B**) Chromatin immunoprecipitation analyses coupled with sequencing (ChIP-seq) analysis was performed with MEF cells and using anti-EBP1 antibody. Overview peak of the EBP1 binding site in the promoter region of HDAC1 gene (upper) is shown. ChIP assay was performed to confirmed EBP1 binding site on HdDAC1 promoter region was analyzed using RT-PCR (bottom). *** *p* < 0.0001. (**C**) MEF cells were subjected to ChIP assay with anti-IgG or RNA polymerase II antibodies to measure the binding of RNA pol II to GAD67 promoter region (upper). Quantification of the binding of RNA pol II to GAD67 promoter region is shown (bottom). *** *p* < 0.0001. (**D**) Luciferase activities of Dnmt1 (left), HDAC1 (middle), and GAD67 (right) were measured using the luciferase assay (Promega). Promoter regions of those genes were cloned using the pGL-4.12 vector and transfected into EBP1 MEF cells shown as *Ebp1*^(+/+)^, *Ebp1*^(+/−)^ or *Ebp1*^(-/-)^. *** *p* < 0.0001. (**E**) Identification of EBP1 binding motif and mutation in the predicted EBP1 binding site in the Hdac1 promoter are shown. Cells were co-transfected using pGL-Hdac1 promoter constructs (WT or mutant) along with the control or *Ebp1* plasmids. *** *p* < 0.0001. All data are shown as the mean ± s.e.m.

Besides Dnmt1, HDAC1, a co-repressor associated with epigenetic regulation, was found increased in the SZ patient brains and was linked to regulation of GAD67 expression in the hippocampus [13,19,29]. We found that HDAC1 and GAD67 expressions are inversely correlated in Ebp1^(+/−)^ mice hippocampus (Figure 5A). HDAC genes were defined as potential targets for EBP1 in chromatin immunoprecipitation analyses coupled with sequencing (ChIP-Seq), The ChIP-Seq data confirmed that EBP1 bind with the HDAC1 promoter region (−430 to −79) (Figure 5B). In the presence of EBP1, RNA polymerase II was barely bound to the HDAC1 promoter region, whereas its promoter binding was increased under conditions of half-depleted level of EBP1 (Figure 5C). Using HDAC1 promoter luciferase reporter plasmid (containing (−430 to −79), we demonstrated that the homozygous depletion of Ebp1 greatly augmented the promoter activity. Heterozygous depletion of EBP1 also enhanced the promoter activity in a dose- dependent manner compared with that of WT-EBP1 expressing cells. However, heterozygous depletion of *Ebp1*, notably ameliorated the GAD67 promoter activity in MEF cells, suggesting that decreased expression of EBP1 can suppress the activity of the GAD67 promoter (Figure 5D). 

Computational analysis of ChIP-seq data predicted a putative EBP1 binding sequences (*p*-Value = 3.7 x 10^-9^) within EBP1 binding region (−430 to −79). To ensure the functional role of EBP1 in *HDAC1* gene regulation, we constructed a mutant *HDAC1* promoter luciferase reporter (containing the (−430 to −79)), in which the putative EBP1 binding sequence (around −278 (TACCA)) contained a mutated randomized sequence (GCGTG). As we expected, EBP1 suppressed WT promoter activity. However, EBP1 overexpression did not interfere with the promoter activity in the presence of mutant promoter, suggesting that this sequence is crucial for EBP1 to meditate the transcriptional repression (Figure 5E). Thus, our data indicates that physiological EBP1 function can alleviate aberrant gene repression caused by altered expression of epigenetic repressors such as HDAC1 and/or Dnmt1.

### 2.6. Loss of Function/Missense Mutation of Ebp1 Gene Decreases GAD67 Expression and Disturbs Epigenetic Control

**Figure 6:**
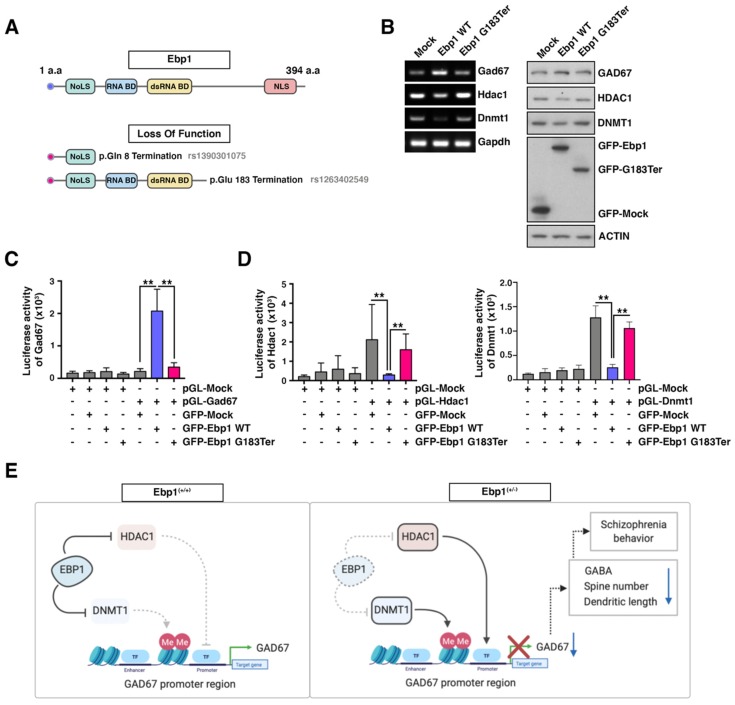
The loss of function/missense mutation of *Ebp1* gene decreases GAD67 expression and disturbs epigenetic control. (**A**) Schematic presentation of two of loss of function mutations, mutation to stop codon, from SZ patients is shown. (**B**) RNA and protein expression levels of Gad67, Hdac1 and Dnmt1 were measured in transfected *Ebp1*^(−/−)^ MEF cells. GFP-mock, Ebp1 WT, or Ebp1 G183Ter constructs were used for transfections. The RNA expression was tested using qRT-PCR with specific primer sets. The protein level was measured using IHC with anti-GAD67, HDAC1. and DNMT1 antibodies. (**C**) *Ebp1*^(−/−)^ MEF cells were co-transfected with pGL-Gad67 promoter along with control or Ebp1 WT, G183Ter mutation construct plasmids. Luciferase activity of GAD67 was measured using the luciferase assay. ** *p* < 0.001. (**D**) Luciferase activity of Hdac1 (left) and Dnmt1 (right) were measured in the transfected Ebp1^(-/-)^ MEF cells which over-expressed Ebp1 WT or G183Ter mutation proteins. ** *p* < 0.001. (**E**) Schematic illustration shows molecular mechanisms of EBP1 in GAD67 epigenetic effects. Created with BioRender. ** *p* < 0.001. All data are shown as the mean ± s.e.m.

Despite very limited information about the role of *Ebp1 gene* in SZ patients, two mutations (the loss of function/mutation to stop codon) have been reported by SZ exome meta-analysis consortium (SCHEMA) (October 2019). One of the mutations was located at the 8th amino acid (c.22C>T; p.Gln8 Ter), suggesting that this mutation may not express functional effects on the EBP1 protein signaling. The other mutation of the EBP1 fragment (c.547 G>T/p.Glu 183 Ter) is located in the area which may or may not influence the functional activity of EBP1 (Figure 6A). Therefore, we constructed the EBP1 repressing fragment (183 amino acids; c.547 G>T/p.Glu 183 Ter) and tested whether this fragment is involved in the development of SZ and abnormal regulation of GAD67. Employing *Ebp1*^(−/−)^ MEF cells, we reintroduced several constructs including GFP-Ebp1-WT, GFP-Ebp1 G183 Ter mutant, or GFP-mock vector into different groups of cells. Reconstitution of EBP1 via GFP-Ebp1-WT transfection suppressed the expression of Dnmt1 and HDAC1, whereas GAD67 expression was upregulated at both the mRNA and protein levels (Figure 6B). However, reconstitution of EBP1 via transfection with GFP- Ebp1 G183 Ter mutant in EBP1 null MEF cells attenuated GAD67 expression but accelerated the expression of DNMT1 and HDAC1. Accordingly, in the presence of GFP- Ebp1 G183 Ter mutant, GAD67 was transcriptionally suppressed, while DNMT1 and HDAC1 escaped from the EBP1-mediated transcriptional repression (Figure 6C,D). In contrast, GFP-WT construct expression led to the transcriptional repression of Dnmt1 and HDAC1 and subsequent upregulation of GAD67. Therefore, this data suggests that EBP1 mutants (at the 8th amino acid or 183 amino acid), which are similar to those discovered in SZ patient, can be associated with the loss of EBP1 protein expression/function and disruption of epigenetic control.

## 3. Discussion

In the current study, we found that heterozygous EBP1-deletion 1 mutant mice exhibited developmental abnormalities in hippocampus, especially in neurons located in SO of CA3/2 region. The mice demonstrated abnormal behaviors including damaged responses to introduction of social novelty, higher levels of anxiety, impaired marble-burying behavior, and other cognitive dysfunctions. These unusual behaviors support the possibility that EBP1 deficient mutant mice presented SZ-like behaviors similar to those observed in SZ models from the previous reports [20,30,31]. The loss of EBP1 resulted in the down regulation of GAD67, in contrast to up-regulation of Dnmt1, and HDAC1 expression levels. Both Dnmt1 and HDAC1 (epigenetic repressors) which are considered to regulate GAD67 expression in the SZ patient, were upregulated in the hippocampus of *Ebp1* mutant mice. Remarkably, EBP1 G183 Ter mutant found in SZ patients did not inhibit the expression of epigenetic repressors, and, thereby, failed to induce GAD67 expression. A significant positive correlation between EBP1 and GAD67 levels has been detected in covariation analysis of human brain transcriptomes. Thus, this data suggested that reasonably high expression of EBP1 is crucial for the GAD67 expression in the hippocampus, while the loss of EBP1 can facilitate development of cognitive and social impairments in SZ patients via deregulation of epigenetic repressors and reduction of GAD67 expression in hippocampus. 

It has been indicated that EBP1 possesses the transcriptional regulatory activity. For instance, short p42 EBP1 isoform was shown to bind with Rb and represses E2F1 mediated transcription in breast cancer cell lines [32]. In HEK293 cells, p48 EBP1 (from *Xenopus* Pa2G4) was detected to interact with transcription factor Six1 and repress transcription activity of Six, although the isoform activated the same reporters in *Xenopus* fibroblast-like cells (XTC-2) [33]. Therefore, EBP1 can regulate either transcriptional repression or activation depending in cell- and tissue-specific manner. Notably, using ChIP-Seq analysis, we provided the direct evidence that EBP1 binds with DNA and modulates gene expression. Specifically, EBP1 binds with the promoter region of *Dnmt1* and represses its expression. Consequently, EBP1 inhibits promoter methylation of the DNMT1 and regulates expression of its target genes, such as *Survivin*. Interestingly, in this study, we found that *HDAC* genes are the potential targets of EBP1. *HDAC* genes contain a putative EBP1 binding sequences within the promoter region which mediates transcriptional repression of *HDAC1* expression by EBP1 (Figure 5). Both Dnmt1 and HDAC1 act as co- repressors of GAD67 mRNA expression via either enhancement of the promoter methylation or suppression of the histone acetylation, respectively. GAD67 expression was decreased in the hippocampus of *Ebp1*^(+/−)^ mice. A similar effect was observed in the postmortem SZ patient brains.

Alternatively, Dnmt1 and HDAC1 expression were upregulated in *Ebp1*^(+/−)^ mice. The effect was accompanied by the distortion of dendrites morphology and decreases in neuronal dendrite size and number in hippocampus (Figure 1 and Figure 2). Considering this data, we questioned whether EBP1 is involved in the regulation of GAD67 transcription via regulation of epigenetic repressors. Indeed, we demonstrated that EBP1 not only suppressed the binding of DNMT1 with the GAD67 promoter, but also directly bonded with HDAC1 promoter region, and, thereby, repressed GAD67 expression. The data indicates that Dnmt1 strongly binds with GAD67 promoter and upregulates HDAC1 expression in hippocampus of *Ebp1*^(+/−)^ mice (Figure 4E and Figure 5A). In agreement with these gene expression patterns, *Ebp1*^(+/−)^ mice showed SZ-like behaviors (Figure 3), indicating the possibility that the functional impairment of EBP1 levels can potentially result in the epigenetic disorder during early brain development. The disorder cannot be compensated by other genes and, consequently, induces developmental cognitive disease such as SZ. 

Epigenetic control is an essential gene-regulation mechanism required for normal neurodevelopment. An independent study suggested that the dysregulation of epigenetic machinery could be involved in etiology of SZ [34]. The expression levels of GAD67 mRNA and protein were found to be reduced in postmortem hippocampal tissues of SZ patients. The effect coincided with the hypermethylation of histone 3 lysine 27 (H3K27), reflecting activation of transcriptional repression and subsequent DNA methylation [35]. Accordingly, Dnmt1 and HDAC1 were found upregulated in the SZ patient brains [12,36]. Although EBP1-dependnent contribution to the etiology of SZ has not been clinically supported yet, unusual upregulation of transcriptional repressors, Dnmt1 and HDAC1, was detected in the hippocampus of *Ebp1*^(+/−)^ mutant mice or in *Ebp1* mutant (c.547 G>T/p.Glu 183 Ter) transfected cells (from SCHMA) with repressed GAD67 expression. This, in turn, reflects that EBP1 is an essential effector that controls neuronal GAD67 levels employing epigenetic repressors. Further investigation is required to confirm the EBP1-linked impact on the development of cognitive diseases. Clarification of mechanisms and roles of epigenetic regulation in cognitive disorders, including SZ, may provide useful therapeutic strategies to restore balanced gene expression.

Chronic NMDA antagonists, phencyclidine (PCP) treatment in rodents has been widely used to model SZ as it displays cognitive defects [37] and reduced PFC activity [38,39]. In particular, subchronic PCP rat models have shown decreased novel object recognition and working memory with social dysfunction while increased repetitive self-grooming and locomotion [40,41,42,43,44,45]. The abnormalities that have been reported in subchronic PCP rat model were similar to those we observed in behavioral deficits in *Ebp1*^(+/−)^ mice. However, *Ebp1*^(+/−)^ mice exhibited a decreased locomotion activity which was increased in subchronic PCP rat model in open field test. In the case of *Ebp1*^(+/−)^ mice, they did not exhibit hyperactivity, but rather displayed anxiety like behaviors. Although we do not clearly understand whether this might be due to the fact that anxiety level simply affects hyperactivity or any other developmental defects could lead to no-hyperactivity, it might be interesting to determine further various aspects of behavioral impairment of *Ebp1*^(+/−)^ mice with other brain regions and neural networks. Functional brain network activity of prefrontal cortex and hippocampus was altered in subchronic PCP rat model [46] and PCP treatment in neonatal mice induced behavioral, histological, and neurochemical abnormalities in adulthood with reduced spine density and reduced GABAergic cells in the hippocampus [47]. Moreover, GAD67 levels were decreased in this model [48,49], though it is not clearly understood whether a molecular mechanism of PCP induced a reduction of GAD 67 level. In the current study, we showed that *Ebp1*^(+/−)^ mice have a reduced volume of whole brain with around 35% smaller hippocampus volume compared with control mice as well as an impaired dendritic development. Along with behavioral and histological deficits in *Ebp1*^(+/−)^ mice, in the hippocampus of *Ebp1*^(+/−)^ mice, both mRNA and protein levels of GAD67 was greatly decreased while DNMT1 and HDAC1, which are associated with low level of GAD67 expression, are up-regulated. Moreover, we demonstrated that EBP1 suppressed the binding of DNMT1 with the GAD67 promoter and directly binds to HDAC1 promoter region, thus leading to repression of GAD67 expression. In recognition of the need to address cognitive dysfunction in patients with SZ and to enhance the development of novel therapies that met clinical need, the development and validation of better animal models for SZ is essential. Subchronic PCP rat model is understood to be a considerably valuable model to produce the negative and cognitive deficits associated with SZ [50]. However, there is some limitation of subchronic PCP rat model validity because this model does not incorporate neurodevelopmental or genetic approaches, which are key to the etiology of SZ in humans. Therefore, since *Ebp1*^(+/−)^ mice displayed a similar cognitive deficits and impaired social behaviors with associated neurochemical changes, further studies of *Ebp1*^(+/−)^ mice could perhaps provide a new use of developmental model for SZ. 

Conclusively, using EBP1-deficient heterozygous mice, we demonstrate a causal relationship between EBP1 functions in brain cells and development of SZ. EBP1 acts as a potent co-regulator of gene expression and coordinates the regulation of epigenetic repressors such as Dnmt1 and HDAC1. Accordingly, the epigenetic repressors were inversely associated with the low level of GAD 67 in EBP1-deficient heterozygous mice (Figure 6E). This work provides a substantial insight into the possible role of the epigenetic machinery in the regulation of the brain architecture and development of neurological diseases.

## 4. Materials and Methods 

### 4.1. Experimental Animals

*Ebp1* knockout mice were generated as described previously [9]. In brief, mouse *Ebp1* gene is located on chromosome 10 (NM_011119) and the gene is composed of 10 exons and extends over 8.4 kb. The *Ebp1* knockout mouse was generated in collaboration with genOway (Lyon, France). Mice were maintained in a controlled environment (12 h light and 12 h dark cycle) and fed a normal chow diet. The gDNA was collected from the mouse ear using the gDNA extraction kit (Sigma Aldrich, Saint Louis, MO, USA). gDNA was genotyped using RT-PCR. This study was reviewed and approved by the Institutional Animal Care and Use Committee (IACUC) of Sungkyunkwan University School of Medicine (SUSM) (code \ SKKUIACUC2018-11-14-2, approval date 16 Nov 2018). SUSM is an Association for Assessment and Accreditation of Laboratory Animal Care International (No. 001004) accredited facility. SUSM strictly follows all the Institute of Laboratory Animal Resources guidelines. All experimental procedures were carried out in accordance with the regulations of the IACUC guidelines at Sungkyunkwan University. 

### 4.2. Antibodies

Anti-Ebp1 (ab186846), anti-Dnmt1 (ab13537), anti-Map2 (ab32454), anti-Tau1 (ab76412), and anti-Psd95 (ab18258) antibodies were acquired from Abcam (Cambridge, MA, USA). Anti-actin (sc-47778), anti-GFP (sc-9996) antibodies were obtained from Santa Cruz Biotechnology (Dallas, TX, USA). Anti-Gad67 (mab5406), anti-Tuj1 (mab1637) and anti-NeuN (abn78) antibodies were acquired from Millipore (Burlington, MA, USA) 

### 4.3. Plasmid Constructs

Human EBP1 was cloned into the pEGFP-C2 vector. PCR-based mutagenesis was performed using the QuikChange site-directed mutagenesis kit (Stratagene, La Jolla, CA, USA) with the following primers: G183Termination forward, 5′–GCACGCCAATATAAGGTATGCT-3′, and reverse, 5′–CAGCATACCTTATATTGGCGTGC-3′. For luciferase assay, promoter region of target genes (Dnmt1, Hdac1 and Gad67) were cloned into pGL-3 vector.

### 4.4. Western Blotting

Transfected cells were washed with PBS and treated with ice-cold lysis buffer (50 mM Tris-Cl, pH 7.4, 150 mM NaCl, 1 mM EDTA, 0.5% Triton X-100, 1.5 mM Na 3 VO 4, 50 mM sodium fluoride, 10 mM sodium pyrophosphate, 10 mM β-glycerolphosphate, 1 mM phenylmethylsulfonyl fluoride, and protease cocktail (Calbiochem, San Diego, CA, USA)). The cell extracts were purified using centrifugation at 20,000× g for 10 min. Proteins were denatured, resolved on SDS-PAGE, and transferred to nitrocellulose membranes (Pall Life Science, Port Washington, NY, USA). The membranes were blocked in 5% skim milk and incubated sequentially with primary antibodies and horseradish peroxidase-conjugated secondary antibodies as described in the manufacturer’s protocols.

### 4.5. Immunohistochemistry (IHC)

All mice were anesthetized deeply with isoflurane and CO_2_ in chamber, following IACUC guidelines at Sungkyunkwan University and the brains were removed, fixed in 4% paraformaldehyde, and incubated with 30% sucrose [51]. The brain was frozen in OCT compound and cut coronally into 20-µm-thick slices. The sections were mounted on slides and washed with ice-cold PBS and permeabilized with 0.25% Triton X-100 in PBS for 1 h, and blocked in 1% bovine serum albumin for 30 min. The cells were immunostained using primary antibodies overnight, and then incubated for 1 h at RT with secondary antibodies (Alexa Fluor 488 for green signal or 546 for red signal.) Nuclei were counter-stained with DAPI stain. Stained tissues were mounted with a mounting medium (Vector Laboratories, Burlingame, CA, USA). Immunostained images were acquired by means of a laser scanning confocal microscope (LSM 710, Carl Zeiss, Oberkochen, Germany). The confocal microscope was controlled by the ZEN software. 

### 4.6. Luciferase Assay 

*Ebp1* MEF cells were plated in culture plates and transfected with 100 ng of GAD67, HDAC1, or Dnmt1 promoter luciferase reporters and 30 ng *Renilla* reporter vector in 6 well plate. Following this, cells were lysed, and luciferase assays were performed using a dual luciferase assay kit (Promega WT, USA) according to the manufacturer’s instruction. The transfection efficiency was normalized against *Renilla* luciferase activity and confirmed transfected genes by immunoblotting. All assays were performed at least in triplicates [52]. 

### 4.7. Isolation of RNA and qRT-PCR

To compare the mRNA levels, quantitative RT-PCR was used. Total RNA was isolated using Takara mini BEST Universal RNA extraction kit (Takara, Japan) from mouse hippocampus. cDNA was prepared from the total RNA using reverse transcription and oligo-dT primers (Takara, Japan). qRT-PCR was performed in triplicates using SsoFast EvaGreen Super Mix (Bio-rad) according to the manufacturer’s instructions. Data is normalized to transcript concentrations using Gapdh for quantitation of the expression of target genes in samples from Ebp1^(+/+)^ and Ebp1^(+/−)^. A total reaction mixture of 20 μL was amplified in a 96-well PCR plate (Bio-Rad). Primers for all target genes are listed in 4.12 primer set table.

### 4.8. Public Dataset Analyses

Gene expression datasets were downloaded from the NCBI Gene Expression Omnibus website (GEO, https://www.ncbi.nlm.nih.gov/geo/query/acc.cgi?acc=GSE15745, public on 20 April 2010) The data with the accession numbers GSE15745 (human temporal cortex and frontal cortex) was used [53]. Differentially expressed genes were assessed using a linear regression method and the R/Bioconductor limma package in R (http://www.r-project.org). [54,55].

### 4.9. ChIP Assay 

A ChIP assay was performed using a ChIP assay kit (cat. 17-259, Millipore, Temecula, CA 92590, USA) according to the manufacturer’s instructions. In brief, a histone was crosslinked to DNA with formaldehyde into the culture dish and glycine was added to quench any unreacted formaldehyde. The cells were scraped into e-tubes and centrifuged at 700× *g* at 4 °C for 1 min. The supernatant was removed, and the cell pellet was resuspended in SDS lysis buffer containing 1× protease inhibitor cocktail. Antibodies, protein G agarose, and chromatin were added for immunoprecipitation and rotated overnight at 4 °C. The protein G agarose-antibody/chromatin complex was washed by resuspending the beads in 1-mL of cold buffers (low salt immune complex wash buffer, high salt immune complex wash buffer, LiCl immune complex wash buffer, and TE buffer in regular sequence). Elution buffer was added into reverse crosslink of the protein/DNA complexes and the mixture was incubated at RT. The mixture was added 5 M NaCl and incubated at 65 °C for 6 h. The purified DNA was collected using spin columns. The immunoprecipitation of target genes were confirmed by immunoblotting with antibodies. Primers from multiple sites relative to TSS designed and pretested in both input and ChIP samples. Only those with singe positive bands in both input and ChIP samples were chosen for the experiment. Purified DNA was subjected to RT-PCR reactions with primers against mouse Gad67 (forward, 5’-AGAGAGCCAACGAGGACACGACC-3’ and reverse, 5’-CACCTCCAGCTGCTTCCTCGTT-3’) and Hdac1 (forward, 5’-CCGGCCTCGAAGCCC-3’ and reverse, 5’-TAGCTGCGTGACCTTGCG-3’) promoter regions.

### 4.10. Methylation-Specific PCR

Genomic DNA was extracted from Ebp1^(+/+)^ and Ebp1^(+/−)^ MEF cells using the Genomic DNA Extraction kit (Sigma). After the digestion with either MSPl or Hpall restriction enzymes using the Epi JET DNA Methylation Analysis kit (Thermo Scientific, Waltham, MA, USA), the endogenous Gad67 promoter was amplified. The Hpall and MSPl restriction enzymes recognized the sites containing CCGG sequence. The Hpall restriction enzyme was unable to cut methylated cytosine, while Mspl cut all CCGG sites. The Gad67 promoter was amplified using a Gad67 promoter-specific primer set. Forward, 5’-GGATCGTGCAAGCAAGGAAG-3’ and Reverse, 5’- TGAGGCAAAGGG-CTGGACAA -3’.

### 4.11. Magnetic Resonance Imaging (MRI)

MRI was performed on a horizontal bore 9.4T/30 cm Burker BioSpec MR system (Billerica, MA, USA) at Neuroscience Imaging Research (IBS) laboratory at the Sungkyunkwan University. The anesthetized animal (1.5% isoflurane in air) was placed in heated cradle where a temperature was sustained at 37 °C. T2-weighted spin-echo images (TR/TE ¼ 5000/50 ms, slice thickness 0.5 mm, 15 slices) were obtained across the entire mouse brain. 

### 4.12. Behavior Test

All behavioral tests were performed using age-matched male mice (8–12 weeks). All behavior studies were performed during day time (lights were left on for 8 h periods). 

Three-chamber social interaction experiments: WT mice (control) were noted t spend more time in the chamber and in close interaction with the novel (introduced) mouse than with the familiar animal. The three-chamber apparatus was placed in a non-transparent plexiglass box (25 × 50 cm) with two transparent partitions that make left, center, and right chambers (25 × 16.7 cm). Each partition has a square opening (5 × 5 cm) in the bottom center areas. A cylindrical wire cage (9 cm diameter; a pencil cup) was used as an inanimate object or the cage was used for the housing of a stranger/new mouse. The scheme of three-chamber social interaction test is displayed (upper). In the first 10 min (left, habituation), test mouse was placed in the center and allowed to explore each chamber as habituation liberally. In the second 10 min (center, sociability), an age- and gender- matched WT B6 new/stranger mouse (mouse 1) was placed in the wired cage of the left chamber. In the last experiment (right, social novelty), a new stranger mouse (mouse 2) was placed in the wired cage of the right chamber. The test mouse was allowed exploring the chamber for 10 min. The movement of the mouse was recorded and analyzed using video tracking software (ToxTrac, Sweden) and Etho Vision XT14 (Noldus, The Netherland).

Novel object recognition test was performed in standard mouse cages. This test consists of three phases, the habituation phase, the familiarization phase and the test phase. In the habituation phase, the mice were habituated to the empty mouse cages to adapt to the environment for 20 min. The next day, in the familiarization phase, mice are placed in the same cage with two identical objects and measured time which a mouse’s nose touched the object or was oriented toward the object and came within 2 cm of it for 10 min. In the test phase, one of the two objects was replaced with a new object as novel, and the test was performed 24 h later for 10 min. The movement of each mouse was recorded and analyzed using video tracking system, EthoVision XT14 (Noldus, The Netherland). 

Open field test was used to assess anxiety and exploratory behaviors. Each individual mouse was placed near the wall-side of the open field area (44.5 × 44.5 cm). The area is separated into two zones and includes the center (28.5 × 28.5 cm) and the periphery zones (around of center zone). Open field test was performed for 20 min. The movement of the mouse was recorded and analyzed automatically using the Animal Activity Meter: Opto-Varimex-5 Auto-Track (Columbus, OH, USA).

Marble-burying test was used to depict anxiety or obsessive-compulsive behaviors. Standard mouse cages were filled with fresh bedding to a depth of 5 cm and 12 marbles (1 cm diameter) were evenly spaced across the bedding. Test mice, WT and Ebp1 heterozygous, were placed in the cage for 30 min and allowed to ambulate liberally. The over 2/3 buried marbles were counted. A photograph was taken of the cage before and after the mouse was kept in the cage. The data was analyzed to determine the average number of marbles buried while the mouse occupied the cage. The experiment was repeated at least three times for each mouse.

Self grooming was observed and recorded in a standard home cage. Mice were individually placed in a home cage and left to roam liberally for 10 min. A mouse behavior was analyzed for 10 min and all grooming-related activities (stroking, scratching of face, head or body with the two forelimbs, or licking body parts) were recorded by an observer blinded to the identity of subject mice. 

Y-maze activity was tested using a symmetrical Y-maze. Each mouse was placed in the side wall of an arm and allowed to explore the maze during a 10 min liberally. The movement of each mouse was recorded and analyzed using Video tracking system, EthoVision XT14 (Noldus, Netherland). 

Passive avoidance is a fear-aggravated test used to evaluate learning and memory. In this test, WT and *Ebp1* mutant mice were allowed to explore the environment for 10 min (600 s) and trained with foot-shock. Afterwards, they had been moved to another box (dark) 3 times on the first day. The next day, those mice were tested and the duration of the freezing time was checked (maximum trial duration = 600 s). The movement of each mouse was recorded and analyzed automatically using Passive/Active Avoidance Box (PACS-30, Columbus Instruments, Columbus, OH, USA). Light was used as the conditioned stimulus. ** *p* < 0.01.

### 4.13. Statistical Analysis

Densitometry analysis of immunoblotting was conducted using Image J software. Image values were normalized to actin bands. Immunohistochemistry images were acquired by ZEN software (ZEISS, Oberkochen, Germany). Data are presented as mean ± S.D. of three independent experiments. Statistical analyses were performed using Stat Graph Prism 8 (Graph- Pad). For statistical significance, experiments with two groups were analyzed using two-tailed Student’s t tests. Data is reported as mean ± SEM. Differences with *p* < 0.05 were considered statistically significant (* *p* < 0.05; ** *p* < 0.01; *** *p* < 0.001); n.s. no statistically significant difference (*p* ≥ 0.05). 

## Supplementary Materials

Supplementary materials can be found at https://www.mdpi.com/1422-0067/21/7/2609/s1. Figure S1. *Ebp1^(+/−)^* mice displayed impaired neural development in stratum oriens (SO) of CA3/2 region in hippocampus. Figure S2. *Ebp1^(+/−)^* mice display SZ-like behaviors. Table S1. List of primers used in qPCR experiments. To compare the mRNA levels, qRT-PCR was used with those primer sets.
