# Supplementary material for: Dysregulation of Epigenetic Control Contributes to Schizophrenia-Like Behavior in Ebp1+/− Mice
Source: Int J Mol Sci. 2020 Apr 9;21(7):2609. doi: 10.3390/ijms21072609 (PMC7178112; doi:10.3390/ijms21072609)
Supplement: Supplementary file 1 [file ijms-21-02609-s001.pdf]

International Journal of Molecular Sciences

MDPI

Table S1. List of primers used in qPCR experiments. To compare the mRNA levels, qRT-PCR was used with those primer sets.

qRT-PCR					
**Gene**	**Primer**	**Sequence**	**Gene**	**Primer**	**Sequence**
Ebp1	Forward	AGA GCA TTT GAA GAT GAG	Gad65	Forward	GGC TCC GGC TTT TGG TCC TT
	Reverse	TCAGTCCCCAGCTTCATT		Reverse	CAG CTT GTT TCC GAT GCC GC
Gad67	Forward	GGT GGA CTG CTC ATG TCC CG	Slc6a1	Forward	GTG GCG GGG CCT TCC TAA TC
	Reverse	GAG CAG CAC GCC CAT CAT CT		Reverse	CCA GCT TCC ATA CGC CCA GG
Dnmt1	Forward	ACG GAA ACC CAA GGA AGA GT	Adcy5	Forward	TGA CCC CAC AAC AGC AGC AG
	Reverse	TTC CGG TCT TGC TTC TCT GT		Reverse	CTG CCA GGC GGA CTT AGA GC
Hdac1	Forward	CGA GAC GGC ATT GAC GAC GA	Gabrg2	Forward	GGT GGA GTA TGG CAC CCT GC
	Reverse	TCC ACA CAC TTG GCG TGT CC		Reverse	CCA GGC TCC TGT TCG GCA AT
Gapdh	Forward	GTG TTC CTA CCC CCA ATG TGT	Gnai2	Forward	GAC CAC GGG CAT CGT GGA AA
	Reverse	ATT GTC ATA CCA GGA AAT GAG CTT		Reverse	TGA CGC CCT CAA AGC AGT GG
Gng3	Forward	ATT GAG GCC AGC TTG TGC CG	Gnao1	Forward	GGA TCG GAT TGG AGC CGG TG
	Reverse	TCT CCC GGA AGG GGT TCT CG		Reverse	CAT AGC CGC TGA GTG CGA CA

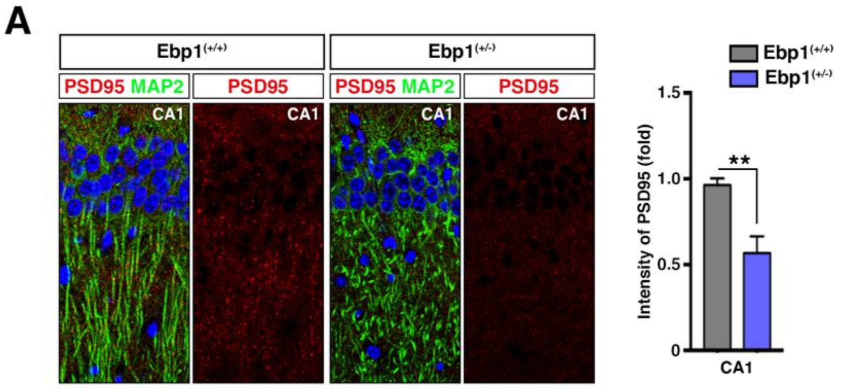

Figure S 1. *Ebp1*$^{(+/-)}$ mice displayed impaired neural development in stratum oriens (SO) of CA3/2 region in hippocampus. (A) Isolated mouse brains from *Ebp1*$^{(+/+)}$ and *Ebp1*$^{(+/-)}$ were frozen, sectioned, and processed using immunohistochemistry with anti-PSD95 (marker for post-synapse, red) and anti-MAP2 (marker for dendrite, green) antibodies. Intensity of PSD95 was measured (right). **$p < 0.001$.

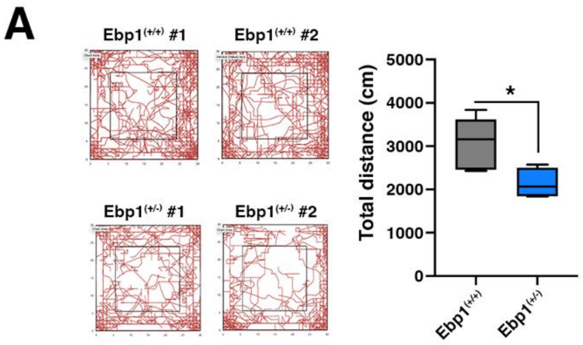

Figure S 2. *Ebp1*$^{(+/-)}$ mice display SZ-like behaviors**.** (A) Open field test was performed for 20 min with *Ebp1*$^{(+/+)}$ and *Ebp1*$^{(+/-)}$ mice. Total movement of the mice were recorded and analyzed automatically with Animal Activity Meter: Opto-Varimex-5 Auto-Track (Columbus, USA).
